# Decorating MOF-Derived Nanoporous Co/C in Chain-Like Polypyrrole (PPy) Aerogel: A Lightweight Material with Excellent Electromagnetic Absorption

**DOI:** 10.3390/ma11050781

**Published:** 2018-05-11

**Authors:** Xiaodong Sun, Xuliang Lv, Mingxu Sui, Xiaodi Weng, Xiaopeng Li, Jijun Wang

**Affiliations:** 1 Key Laboratory of Science and Technology on Electromagnetic Environmental Effects and Electro-Optical Engineering, The Army Engineering University of PLA, Nanjing 210007, China; xiaodongsun1001@hotmail.com (X.S.); xllu1957@126.com (X.L.); plasmx@126.com (M.S.); 2 PLA Rocket Force Research Institute, Beijing 100011, China; fefebi@163.com; 3 School of Information and Communications, National University of Defense Technology, Xi’an 710106, China; 4 Research Institute for National Defense Engineering of Academy of Military Science PLA China, Beijing 100036, China

**Keywords:** zeolite imidazole framework, electromagnetic absorption, interfacial polarization, heterostructure

## Abstract

To clear away the harmful effects of the increment of electromagnetic pollution, high performance absorbers with appropriate impedance matching and strong attenuation capacity are strongly desired. In this study, a chain-like PPy aerogel decorated with MOF-derived nanoporous Co/C (Co/C@PPy) has been successfully prepared by a self-assembled polymerization method. With a filler loading ratio of 10 wt %, the composite of Co/C@PPy could achieve a promising electromagnetic absorption performance both in intensity and bandwidth. An optimal reflection loss value of −44.76 dB is achieved, and the effective bandwidth (reflection loss lower than −10 dB) is as large as 6.56 GHz. Furthermore, a composite only loaded with 5 wt % Co/C@PPy also achieves an effective bandwidth of 5.20 GHz, which is even better than numerous reported electromagnetic absorption (EA) materials. The result reveals that the as-fabricated Co/C@PPy—with high absorption intensity, broad bandwidth, and light weight properties—can be utilized as a competitive absorber.

## 1. Introduction

The utilization of electromagnetic waves has been expanded extensively in both civilian and military fields, yet it generates potential hazards on the performance of sophisticated electronic devices and biological immune systems of human beings and wildlife [1,2,3,4]. Tremendous efforts have been devoted in recent decades toward investigating suitable electromagnetic absorption (EA) materials to eliminate or at least decrease these ensuing problems [5,6,7].

Traditional absorbers are mainly composed of ferromagnetic metals [8], carbon [9], and conducting polymers materials [10]. However, ferromagnetic metals are susceptible to corrosion, and are high density with insufficient bandwidth [11]; the high conductivity of the pure carbon and conducting polymers materials always induce an eddy current effect and reflection [12]. In general, the attenuation capacity and the complementarity between the complex permittivity and complex permeability (namely, impedance matching) are two crucial factors in the design of outstanding absorbers [13,14,15]. Some requirements including wide effective bandwidth (reflection loss lower than −10 dB), being lightweight, and low thickness should also be satisfied [16,17,18]. Specifically, the development of functional dielectric materials modified with other composites can represent a significant advance to improve EA performance both in intensity and width. Taking polypyrrole (PPy) as an example, it is a type of the intrinsically conducting polymers which has been investigated as a permittivity regulation for EA materials mainly result from its dielectric property [19,20]. Furthermore, the lightweight of PPy aerogel after drying out the solvent which also matches the feature of high-performance absorbers. So far, substantial efforts have been devoted to exploiting PPy based materials for EA advances and the exhibited performances are highly desirable, such as Fe_3_O_4_/PPy/PANI [21], PPy/RGO (reduced graphene oxide) [12], CIP (carbonyl iron powder)@PPy [22], γ-Fe_2_O_3_/PPy [23], and PPy/SiC [24].

Apart from the aforementioned absorbers, recently, metal-organic frameworks (MOFs) have been the focus of intense research due to their wide spectrum of useful characteristics [25,26,27,28]. The structure of MOFs is comprised of metal ions or clusters that are connected by electron-donating “linker” groups to create a networked structure with well-defined pores [29,30,31]. In the past few years, MOFs have been intensively used as excellent carbon precursors to synthesize nanoporous carbon materials via direct carbonization without using auxiliary templates [32]. Especially, the previous reports suggested that Co/C composites (the resultant of a typical zeolite imidazole framework, ZIF-67 [Co(2-methylimidazole)_2_]) or their hybrids have significant potential for a lightweight EA material due to their increased permittivity and high porosity [33]. For example, Liang and coworkers obtained selectively nanoporous carbon materials consisting of ZnO/NPC@Co/NPC as the shells by the thermal treatment of ZIF-8@ZIF-67 crystals [26]. The composite ZnO/NPC@Co/NPC-0.5 sample filling with 50 wt % of paraffin shows a maximum reflection loss (RL) of −28.8 dB at a thickness of 1.9 mm. A functionalized Co/C composite has also been synthesized by Qiang et al. via in situ pyrolysis of ZIF-67 [34]. Under the optimum conditions, Co/C-800 as the best candidate displays excellent EA property, which the minimum RL can even reach up to −39.6 dB at a thickness of 2.0 mm. It is expected that impedance matching degree and attenuation capability can be adjusted simultaneously by the combination of the Co/C composites and other absorbers. Note that neither extremely high nor low permittivity can promote the formation of high-performance absorber, and it is crucial to take both impedance matching and attenuation capacity into account. Herein, a chain-like PPy aerogel decorated with MOF-derived nanoporous Co/C (Co/C@PPy) has been successfully prepared by a self-assembled polymerization method. We hope that the combination of these components could result an appropriate permittivity value, thus lead a high EA performance simultaneously. The approach is quite facile (only several minutes) and convenient for mass production. Taking advantage of the heterostructure constructed by ternary components, a proper match behavior and the induced intensified interfacial polarization have been achieved. To best of our knowledge, such material has rarely been reported to date. As a result, this hybrid aerogel exhibits the feature of lightweight, wide effective EA bandwidth and strong attenuation capacity with an extremely low filler loading ratio simultaneously, which meets the standards of an absorber. From this view, the Co/C@PPy aerogel fabricated in our work is promising for practical applications as a high-performance absorber.

## 2. Materials and Methods 

### 2.1. Materials

All regents were of analytical grade and used without further purification. Cobalt nitrate hexahydrate (Co(NO_3_)_2_·6H_2_O), ferric chloride (FeCl_3_·6H_2_O), 2-methylimidazole (mIM), methanol, ethanol, and the pyrrole monomer were purchased from GENERAL-REAGENT, Titan Scientific Co., Ltd., Shanghai, China. Deionized water was obtained from Direct-Q3 UV, Millipore (Burlington, MA, USA).

### 2.2. Synthesis of Nanoporous Co/C Composites

Porous Co/C composites were prepared by the calcination of ZIF-67 polyhedron precursors under Ar. ZIF-67 polyhedron were prepared by the simple precipitation as reported previously [34]. In a typical procedure, 0.6 g Co(NO_3_)_2_·6H_2_O and 0.8 g mIM dissolved in a methanol solution (55 mL) to generate a purple suspension at room temperature. After vigorous magnetic stirring for 6 h, the resulting solution was aged for another 24 h and then centrifuged, rinsed with methanol, and dried at 45 °C for 12 h. To convert ZIF-67 to nanoporous Co/C nanoparticles, the as-fabricated ZIF-67 was transferred to a combustion boat and heated at a rate of 2 °C·min^−1^ and maintained at 600 °C for 6 h.

### 2.3. Synthesis of Synthesis of Co/C@PPy Aerogel

Briefly, 135.0 mg pyrrole monomer and 12.0 mg Co/C nanoparticles were slowly dissolved in a mixture containing 2.0 mL deionized water and 2.0 mL absolute ethanol. Then 1.6 g FeCl_3_·6H_2_O ultrasonically dispersed in 1.0 mL deionized water and 1.0 mL absolute ethanol was stepwise injected into the above solution under mechanical stirring for 5 min and the hydrogel was obtained by aging for 24 h. The precipitate was filtered, washed, and dialyzed several times with deionized water and ethanol to wipe out impurities, and then dried at 50 °C for 12 h to form an aerogel.

### 2.4. Characterization and Measurement

The structural analyses of the composites prepared in this work were obtained by X-ray diffraction (XRD) using a X-ray diffraction (D8-Advance, Bruker, Germany) equipped with Cu-Kα radiation (1.5406 Å). The morphology, size distribution and shape of the composites were observed with a transmission electron microscope (TEM), high resolution TEM (HRTEM) by using a field emission TEM (JEM-2100F, JEOL, Tokyo, Japan) and scanning electron microscope (SEM) by using a field emission scanning electron microscope (FE-SEM, S4800, Hitachi, Tokyo, Japan). Raman spectroscopy was carried out via Raman microscope (Renishaw, London, UK) equipped with an excitation line of 532 nm. X-ray photoelectron spectra (XPS) were recorded using a ESCALAB 250Xi X-ray photoelectron spectrometer (Thermo Fisher Scientific, Waltham, MA, USA) equipped with a monochromatic Al Kα X-ray source (1486.6 eV). The thermogravimetric analysis were carried out on a SDT Q600 TGA (TA Instruments, Newcastle, DE, USA). The details on the electromagnetic measurements of the samples have been described elsewhere. The complex relative permeability (*μ_r_* = *μ*′ − *jμ*″) and permittivity (*ε_r_* = *ε*′ − *jε*″) were calculated from the S-parameters tested by a vector network analyzer (VNA, N5242A PNA-X, Agilent, Agilent Technologies Inc., Santa Clara, CA, USA). The theoretical reflection loss (RL) can be calculated based on the *ε_r_* and *μ_r_* at a given frequency and layer thickness by means of the equations [35,36,37]

(1) $$\mathrm{RL}=20\;\log|\left(Z_{in}-Z_{0}\right)/\left(Z_{in}+Z_{0}\right)|$$

(2) $$Z_{in}=Z_{0}\sqrt{\frac{\mu_{r}}{\varepsilon_{r}}}\tanh\left(j\frac{2\pi fd\sqrt{\mu_{r}\varepsilon_{r}}}{c}\right)$$

where *Z_in_* is the input characteristic impedance, *c* is the velocity of light in vacuum, *f* is the frequency of the incident wave, *d* is the thickness of the composites, *Z*_0_ = 376.7 Ω is the intrinsic impedance of free space. In general, materials with RL value of less than −10 dB (comparable to 90% attenuation) are considered as suitable absorbers.

## 3. Results and Discussion

### 3.1. Characterization of Samples 

**Figure 1:**
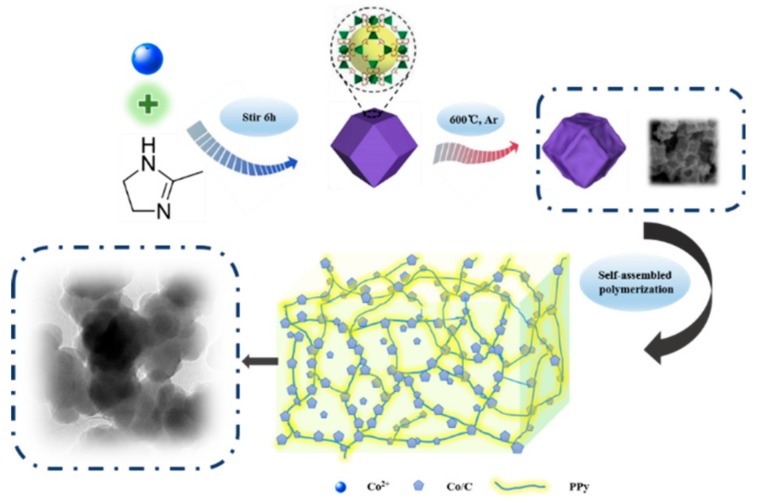
Schematic representation of the formation of Co/C@PPy aerogel.

Figure 1 discusses the schematic representation for the formation of Co/C@PPy. In brief, via a process of oxidation polymerization, Co/C nanoparticles are uniformly embedded in a three-dimensional network structure constructed by PPy chains. With a sufficient filler loading of Co/C@PPy, the conductive network can be formed spontaneously. When the incident electromagnetic wave gets into such network, the electromagnetic wave energy is transferred in the form of microcurrent and consumed. In this work, not only the multiple reflection in the matrix but also the interfacial polarization induced by the interfaces between Co, C, PPy, paraffin matrix and air bubbles play a significant role in enhancing the EA property.

**Figure 2:**
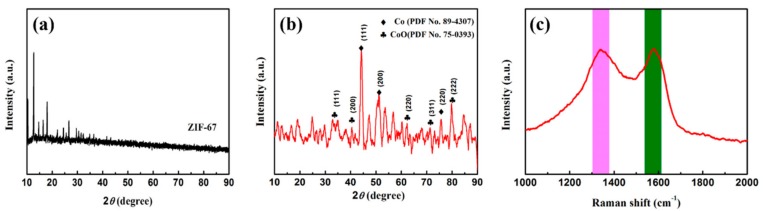
XRD patterns of ZIF-67 (**a**); Co/C (**b**) and Raman spectra of Co/C (**c**).

XRD results are investigated to ascertain the constituents of the ZIF-67, Co/C, and Co/C@PPy, and the spectra are presented in Figure 2 and Figure S1. The positions of the diffraction peaks of the obtained ZIF-67 corresponded to the XRD pattern simulated from the single crystal data of ZIF-67 [34]. As for Co/C in Figure 2b, the main peaks observed at 2θ values of 44.5°, 51.3°, and 75.9° can attributed to (111), (200), and (220) planes of metallic Co according to JCPDS No. 89-4307. Some weak diffraction peaks of 37.7°, 42.6°, 62.2°, 74.2°, and 78.1° (labeled as “♣”) assigned to CoO (JCPDS no. 75-0393) and the corresponding crystal planes are (111), (200), (311), (222). The existence of CoO can be attributed to the exposure of small amount of Co particles to air. Because Co is a moderately reactive element, the deposited Co nanoparticles can be slowly oxidized [25]. In addition, the absence of corresponding diffraction sharp peak for graphite at around 26° suggests the homogeneous dispersion on the surface of the nanoporous Co/C composites [38]. PPy shows an amorphous structure since the broad XRD pattern is around 20 to 30° (Figure S1) [20]. Raman spectroscopy is well known as a powerful tool for the characterization of graphite based materials. In Figure 2c, the Raman spectra of Co/C composite is associated with a typical D band and G band at around 1338 cm^−1^ and 1579 cm^−1^, respectively. The D band becomes active in perfect graphite, which corresponds to the breathing mode of k-point photons of A_1g_ symmetry, and the G band is ascribed to the vibration of sp^2^ carbon atoms [39]. The intensity ratio of D band and G band (I_D_/I_G_) is a criterion to evaluate the graphitization degree of graphitic materials [40]. In this situation, the value of I_D_/I_G_ is 1.1 for Co/C, and such high value is mainly derived from the lower graphitization degree, which is near identical to the broad and weak diffraction peaks at around 26° in the XRD pattern.

**Figure 3:**
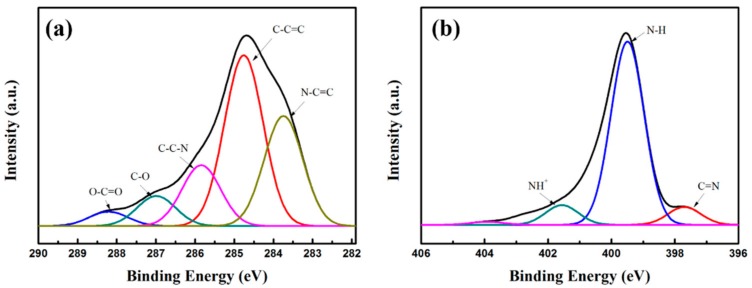
XPS spectra of Co/C@PPy: (**a**) N 1s; (**b**) C 1s.

**Figure 4:**
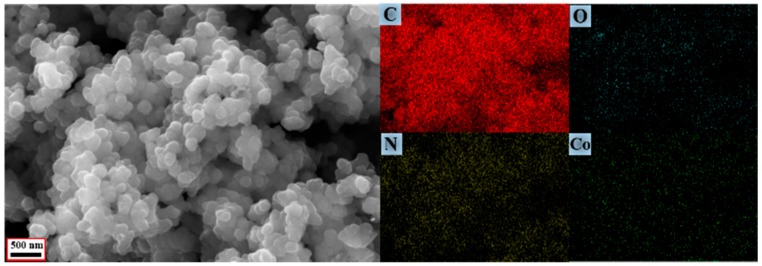
SEM image of the fracture section of the Co/C@PPy and corresponding elemental mappings of C, O, N and Co.

Surface elemental chemical states are confirmed by XPS measurement and the results are shown in Figure 3. In Figure 3a The C 1s peaks can be resolved into five rational Gaussian peaks at 283.55, 284.61, 285.86, 287.05, and 288.31 eV, which can be assigned to the structures of N–C=C, C–C=C, C–C–N, C–O, and O–C=O, indicating the existence of carbonyl defects in PPy [19]. The N 1s peaks presented in Figure 3b is decomposed into three Gaussian peaks with 401.55, 399.45, and 397.70 eV, respectively, which are corresponded to positively charged nitrogen atoms (NH^+^), secondary amine-like structure (N–H), and imine-like structure (C=N) of pyrrole ring. Besides, SEM characterization of the fracture section of the Co/C@PPy and corresponding elemental mappings of C, O, N, and Co are displayed in Figure 4. It is demonstrated that Co nanoparticles possess a relatively uniform distribution in the carbon matrix.

**Figure 5:**
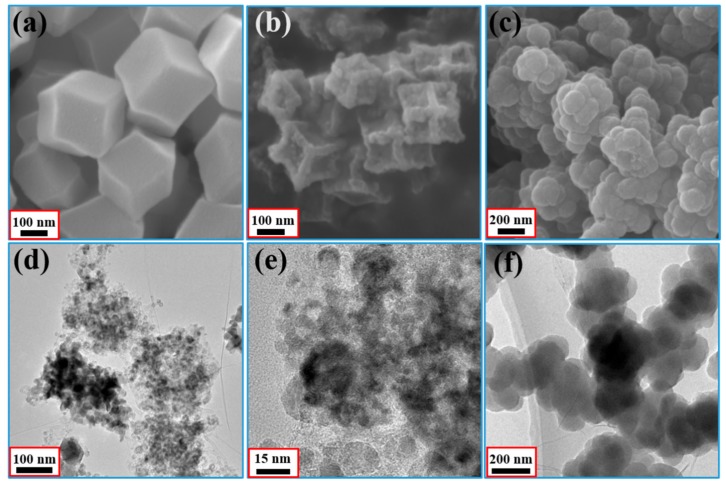
SEM images of ZIF-67 (**a**); Co/C (**b**) and Co/C@PPy (**c**); TEM images of Co/C (**d**,**e**); and Co/C@PPy (**f**).

The morphologies and nanostructures of the samples prepared in this study are characterized by using SEM and TEM. Figure 5a displays some rhombic dodecahedrons which belong to ZIF-67 polyhedron, and the average diameter of ZIF-67 polyhedron is about 300 nm. SEM image of the porous Co/C composites which prepared by the calcination of ZIF-67 precursors is presented in Figure 5b. The corresponding TEM images with different magnification are also presented in Figure 5d,e. It can be observed from Figure 5b that the composites still possess a rhombic dodecahedral shape after calcination process, while the average diameter shows an obvious decline from ~230 nm to ~180 nm. This is because the high temperature consumes the organic components, leading the crystals shrink to a large extent and exhibit a sunken surface. Co/C@PPy with chain-like structure is identifiable from Figure 5c,f. Especially, XRD pattern presented in Figure S1 shows that the intensities of the diffraction of Co or CoO are greatly reduced owing to the presence of PPy with its amorphous nature. This interesting phenomenon is consistent with the morphology presented in Figure 5e, the detailed morphology of Co/C can hardly be observed because of the heavy PPy coating.

### 3.2. Electromagnetic Absorption Property

**Figure 6:**
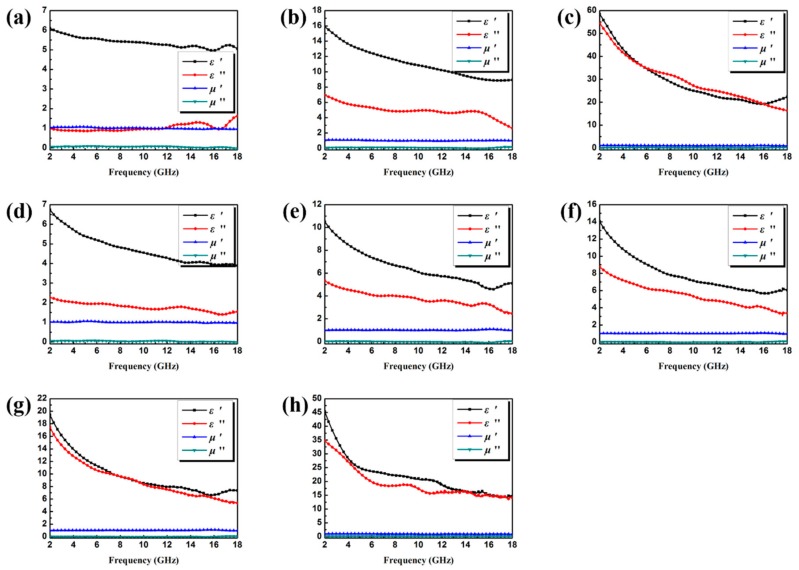
Frequency dependence of real and imaginary parts of complex permittivity and permeability of Co/C with the filler loading of 15 wt % (**a**), 30 wt % (**b**), 45 wt % (**c**); and Co/C@PPy with the filler loading of 5 wt % (**d**), 10 wt % (**e**), 12 wt % (**f**), 15 wt % (**g**), 30 wt % (**h**).

Electromagnetic attributes (*μ_r_* and *ε_r_*) of Co/C and Co/C@PPy loaded various filler are tested and shown in Figure 6. Generally speaking, the real parts of the electromagnetic attributes (*ε*′, *μ*′) are correlated with the amount of polarization in the composite and represent the storage ability of the electric and magnetic energy. While the imaginary parts (*ε*″, *μ*″) denote the dissipated electric and magnetic energies. Because of the rare ratio of the magnetic constituents in these samples, the values of *μ*′ and *μ*″ are approximately constant (*μ*′ ≈ 1.0 and *μ*″ ≈ 0) with a slight fluctuation, and it is suggested that all these samples belong to dielectric loss absorbers. Generally, in the frequency range of microwaves, dielectric loss mainly derives from the dipolar polarization and interfacial polarization. In this work, the dipolar polarization can be originated from carbon and PPy aerogel, while the interfacial polarization mainly provided by the the heterostructure constructed by ternary components. Based on the equation *ε*′ = *ε*″/2*πf* + *ε*_∞_ [5], the variation trend of *ε*′ remains similar to that of *ε*″. As shown in Figure 6, all the samples present typical frequency dependent permittivity. Specifically, the values of *ε*′ decrease with the increasing frequency which mainly result from the fact that in the high GHz frequency region, the dielectric polarization fails to catch up with the variable electromagnetic field. With the increase of Co/C loading (from 15 wt % to 45 wt %), significant enhancements are achieved in both *ε*′ and *ε*″ (Figure 6a–c). The increment of *ε*′ can be attributed to the sufficient conductive interconnections. Besides, the enhanced storage capability and dissipation ability are also indicated. However, it is known that a proper value of permittivity is beneficial for impedance matching. Note that the addition of PPy (Co/C@PPy) noticeably improves the permittivity. The conductive interconnections could be established even in an extremely low filler loading ratio (Figure 6d), which may indicate the dielectric property of Co/C are highly enhanced by the PPy aerogel.

**Figure 7:**
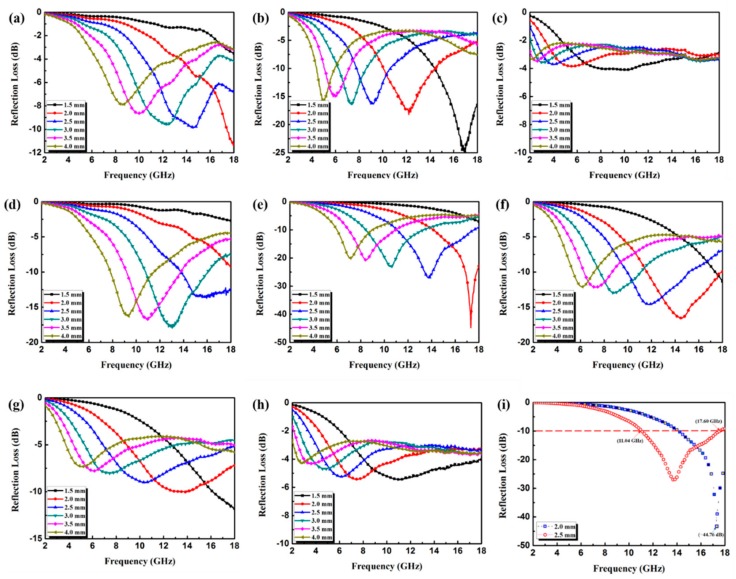
Reflection loss curves of paraffin composites containing 15 wt % (**a**), 30 wt % (**b**), and 45 wt % (**c**) Co/C, respectively; reflection loss curves of paraffin composites containing 5 wt % (**d**), 10 wt % (**e**), 12 wt % (**f**), 15 wt % (**g**), and 30 wt % (**h**) Co/C@PPy, respectively; Reflection loss curves of paraffin composites containing 10 wt % (**i**) under the thickness of 2.0 mm and 2.5 mm. The testing frequency range is from 2 to 18 GHz.

Figure 7a–c depicts the simulated reflection loss (RL) curves obtained for Co/C paraffin composites with Co/C loading of 15–45 wt % at various thickness (1.5–4 mm). Note that the 30 wt % Co/C paraffin composite exhibits the best EA performance. Specifically, the minimum RL of −24.98 dB is achieved at 16.96 GHz with the thickness of 1.5 mm. The broadest effective bandwidth can reach 4.4 GHz (10.08–14.48 GHz) when the thickness is 2.0 mm. In the low filler loading (15 wt %). Because of the high dispersion of the Co/C composite in the paraffin matrix, the conductive interconnections can hardly be formed. While in the high filler loading (45 wt %) condition, the extremely high values of *ε*′ and *ε*″ cause the impedance mismatch and thus lead the electromagnetic wave reflect on the surface rather than absorption. The aforementioned reasons may explain the bad EA performance. Obviously, the EA properties of Co/C@PPy are substantially enhanced relative to the pristine Co/C. It can also be found that a higher concentration in this material leads to opposite EA performance. As shown in Figure 7d, the Co/C@PPy composite with a loading of only 5 wt % even shows a strong absorption both in intensity and bandwidth. The optimal RL is up to −17.85 dB at 12.92 GHz with the thickness of 3.0 mm, corresponding to an effective bandwidth of 5.2 GHz (10.84–16.04 GHz). It is rare to find other reported materials which can exhibit such a broad EA bandwidth under this extreme filler loading. Furthermore, when the filler loading is up to 10 wt % (Figure 7e), more Co/C@PPy molecules connect with each other and an efficient conductive network is formed. In addition, the proper complex permittivity also leads a better impedance match. The optimal RL value of −44.76 dB can be achieved at 17.32 GHz with the thickness of 2.0 mm. A considerable broad effective bandwidth of 6.56 GHz (11.04–17.60 GHz) is reached with the thickness of 2.5 mm (As shown in Figure 7g). The result can fully demonstrate that the as-prepared Co/C@PPy is much more efficient than that of most other ZIF-67 derived Co/C composites or their hybrids. Herein, take the work presented by Lü et al. [25] as an example, the exhibited maximum reflection loss of Co/C-500 can reach −35.3 dB at 5.8 GHz with a thickness of 4 mm, the effective absorption bandwidth is 5.80 GHz with a thickness of 2.5 mm, and the corresponding filler loading ratio is as large as 40 wt %. By comparison, the as-prepared Co/C@PPy has advantages in intensity, bandwidth, matching thickness, as well as efficient filler loading. The excellent EA performance of Co/C@PPy may be attributed to the following fact. First, the unique chain-like PPy aerogel and the porous feature of Co/C itself can induce more multiple reflection and diffuse scattering of the incident electromagnetic wave. Second, the multi-interfaces between Co/C and PPy would induce interfacial polarization, are equally important for the attenuation.

**Figure 8:**
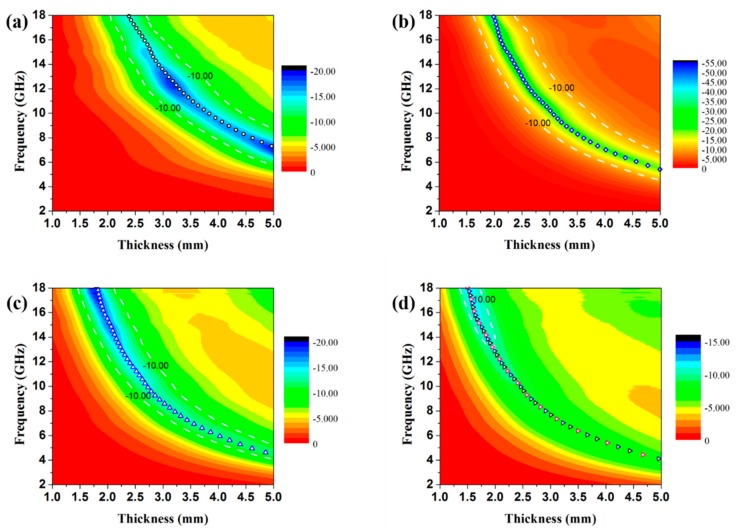
Contour maps of reflection loss of the paraffin composites containing 5 wt % (**a**), 10 wt % (**b**), 12 wt % (**c**), and 15 wt % (**d**) Co/C@PPy and the calculated curves of *t*_m_ according λ/4 model, respectively. The testing frequency range is from 2 to 18 GHz. (white line: region of −10 dB).

One phenomenon can be observed that all the samples achieve the minimum RL value at a certain thickness, and all their RL peaks shift to lower frequency with the increasing thickness. To illustrate why the RL peaks appear at these certain thicknesses, the simulations of the matching thickness versus the achieved peaks frequency are completed based on the equation: *t*_m_ = *nc*/(4*f*_m_$\sqrt{\left|\varepsilon_{r}\right|\left|\mu_{r}\right|}$) [22]. When the matching thickness satisfies the equation, then the reflected wave and incident wave will be out of opposite phase, resulting in the dissipation at the interface. In Figure 8, the 2D contour maps of reflection loss of the paraffin composites containing different loading ratio of Co/C@PPy as well as the corresponding calculated curves of *t*_m_ according *λ*/4 condition are plotted. The points of the minimum RL lie on the curves of *t*_m_ for each sample. So the result demonstrates that the EA activities of the as-prepared samples obey the quarter-wavelength principle.

**Figure 9:**
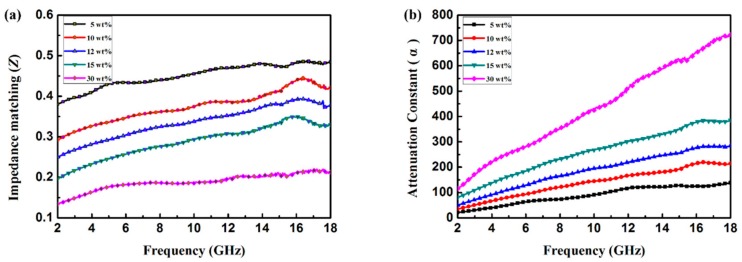
Frequency dependence of impedance matching ratio (**a**) and attenuation constant (**b**) for paraffin composites containing different loading ratio of Co/C@PPy.

Generally, impedance matching ratio *Z* calculated by *Z* = *Z_in_*/*Z*_0_ can reveal the degree of impedance matching. If *Z* = 1, namely the incident wave can enter into the composite entirely with zero-reflection on the surface [39]. A higher *Z* value implies the preferable EA property. In addition, the energy attenuation should also be taken into account. The attenuation constant (*α*) can be expressed as [8]

(3) $$\alpha =\frac{\sqrt{2}\pi f}{c}\times \sqrt{\left(\mu^{”}\varepsilon^{”}-\mu^{\prime }\varepsilon^{\prime }\right)+\sqrt{{\left(\mu^{”}\varepsilon^{”}-\mu^{\prime }\varepsilon^{\prime }\right)}^{2}+{\left(\mu^{\prime }\varepsilon^{”}+\mu^{”}\varepsilon^{\prime }\right)}^{2}}}\;$$

where *c* is the velocity of light in a vacuum. In Figure 9, the calculated impedance matching ratio (*Z*) and the attenuation constant (*α*) for paraffin composites containing different loading ratio of Co/C@PPy are plotted. An excellent absorber should consider both attenuation ability and impedance matching at the same time rather than unilateral superior performance. From Figure 9, it can be found that the composite loaded with 10 wt % has the high Z value as well as a suitable attenuation constant, which are in favorable for the enhancement of EA property. Table S1 shows the EA properties of some recently reported carbon derived-MOF or PPy based composites. In comparison with these composites, the chain-like structured Co/C@PPy takes the advantages in the bandwidth and intensity. Furthermore, the extremely low filler loadings (only 5 wt % and 10 wt %) make Co/C@PPy more competitive in application. The excellent EA property of the as-prepare Co/C@PPy mainly benefit from its moderate impedance matching, strong attenuation capacity, as well as the multiple reflection induced by the unique structure.

## 4. Conclusions

In summary, a chain-like structured PPy aerogel decorated with MOF-derived nanoporous Co/C (Co/C@PPy) has been successfully prepared and its EA property was firstly investigated. In the unique ternary composite, the additional interfaces can induce the interfacial polarization, which further contributes to the reflection loss. Compared to pristine MOF-derived nanoporous Co/C, Co/C@PPy exhibits substantially enhancement with high intensity and broad width at a low thickness and an extremely low filler loading. The composite loaded with 10 wt % Co/C@PPy can reach the optimal RL value of −44.76 dB at 17.32 GHz with the thickness of 2.0 mm. A considerable broad effective bandwidth of 6.56 GHz (11.04–17.60 GHz) is achieved with the thickness of 2.5 mm. A composite only load with 5 wt % Co/C@PPy also shows an effective EA bandwidth of 5.20 GHz, which is even better than numerous reported EA materials. The outstanding performance can be attributed to a proper impedance matching and a high dielectric loss. Besides, the unique chain-like PPy aerogel and the porous feature of Co/C itself can induce more multiple reflection and scattering of the incident electromagnetic wave, and are equally important for the attenuation. Therefore, it is believed that the Co/C@PPy aerogel with high absorption intensity, broad bandwidth, and light weight can be utilized as a competitive absorber.

## Supplementary Materials

The following are available online at http://www.mdpi.com/1996-1944/11/5/781/s1, Figure S1: XRD patterns of Co/C@PPy, Figure S2: Elemental mappings of C and Co for Co/C composites, Figure S3: TG curves of ZIF-67 under air and Ar atmosphere, Table S1: EA performance of typical MOF-derived Co/C or PPy based composites reported in this work and recent literatures.
