# Supplementary material for: Decorating MOF-Derived Nanoporous Co/C in Chain-Like Polypyrrole (PPy) Aerogel: A Lightweight Material with Excellent Electromagnetic Absorption
Source: Materials (Basel). 2018 May 11;11(5):781. doi: 10.3390/ma11050781 (PMC5978158; doi:10.3390/ma11050781)
Supplement: Supplementary file 1 [file materials-11-00781-s001.pdf]

materials

Supplementary

Decorating MOF-Derived Nanoporous Co/C in Chain-Like Polypyrrole (PPy) Aerogel: A Lightweight Material with Excellent Electromagnetic Absorption

Xiaodong Sun, Xuliang Lv, Mingxu Sui, Xiaodi Weng, Xiaopeng Li, and Jijun Wang

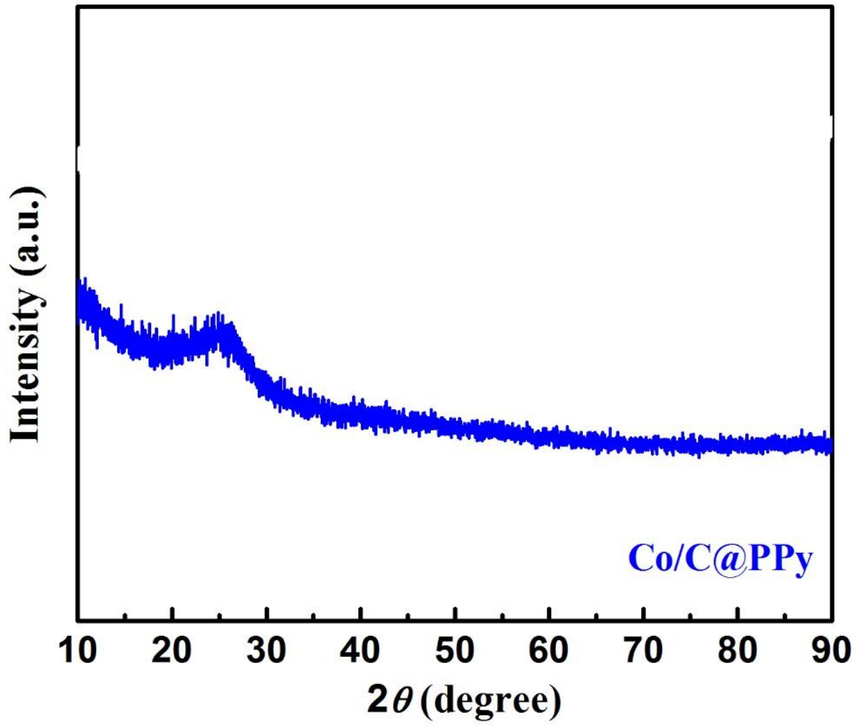

Figure. S1 XRD patterns of Co/C@PPy.

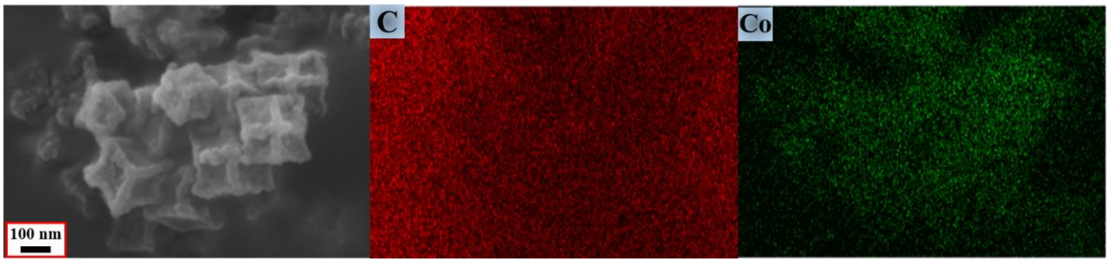

Figure. S2 Elemental mappings of C and Co for Co/C composites.

To well understand the formation process of Co/C composites during the heat treatment, the precursor, ZIF-67, are thermally analyzed by TGA in air and Ar atmosphere in the temperature range of 0 - 700°C with a heating rate of 10°C min^{-1}. For the curve tested under air atmosphere, the decline indicates that ZIF-67 underwent a drastic weight loss between 310°C and 490°C. No further weight loss occurred indicates that the complete decomposition of the organic ligands in ZIF-67. When

subjected to heat treatment under Ar atmosphere, the relative low weight loss in decomposition (20.5%, 400°C -600°C) mainly results from the generation of carbon materials.

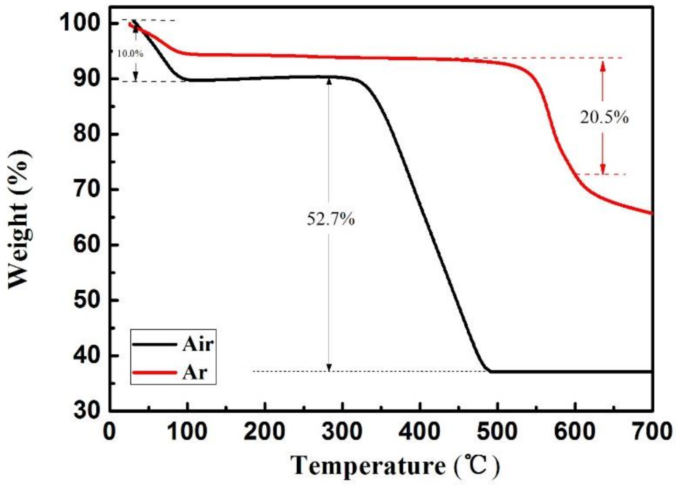

Figure. S3 TG curves of ZIF-67 under air and Ar atmosphere.

Table S1 EA performance of typical MOF-derived Co/C or PPy based composites reported in this work and recent literatures (NPC: nanoparticles; CIP: carbonyl iron powder; Z-BCF:Z-type barium ferrite).

Composite	(wt%)	Thickness (mm)	bandwidth (GHz)	Optimal RL (dB)	Ref.
Co/C/MWCNTs	15	1.10	~2.50	−48.90	[1]
Co/C	60	2.50	5.80	−35.30	[2]
Co/C	30	2.00	3.80	−39.60	[3]
ZnO/NPC@Co/NPC	50	1.90	4.20	−28.80	[4]
CIP@PPy	33	2.20	6.10	−39.50	[5]
Co/PPy	30	2.00	4.77	−33.00	[6]
Fe_3O_4/PPy	30	1.70	4.00	−35.70	[7]
PPy/carbon	40	3.00	1.09	−38.10	[8]
(Z−BCF/SiO_2)@PPy	66.67	2.00	5.06	−19.65	[9]
Co/C@PPy	5	3.00	5.20	−17.85	This
Co/C@PPy	10	2.00	6.56	−44.76	work

References

1. Yin, Y.; Liu, X.; Wei, X.; Li, Y.; Nie, X.; Yu, R.; Shui, J. Magnetically Aligned Co-C/MWCNTs Composite Derived from MWCNT-Interconnected Zeolitic Imidazolate Frameworks for a Lightweight and Highly Efficient Electromagnetic Wave Absorber. *ACS Appl. Mater. Interfaces* **2017**, *9*, 30850–30861.
2. Lü, Y.; Wang, Y.; Li, H.; Lin, Y.; Jiang, Z.; Xie, Z.; Kuang, Q.; Zheng, L. MOF-Derived Porous Co/C Nanocomposites with Excellent Electromagnetic Wave Absorption Properties. *ACS Appl. Mater. Interfaces* **2015**, *7*, 13604–13611.
3. Qiang, R.; Du, Y.; Chen, D.; Ma, W.; Wang, Y.; Xu, P.; Ma, J.; Zhao, H.; Han, X. Electromagnetic functionalized Co/C composites by in situ pyrolysis of metal-organic frameworks (ZIF-67). *J. Alloys. Compd.* **2016**, *681*, 384–393.
4. Liang, X.; Quan, B.; Ji, G.; Liu, W.; Cheng, Y.; Zhang, B.; Du, Y. Novel nanoporous carbon derived from metal-organic frameworks with tunable electromagnetic wave absorption capabilities. *Inorg. Chem. Front.* **2016**, *3*, 1516–1526.
5. Sui, M.; Lü, X.; Xie, A.; Xu, W.; Rong, X.; Wu, G. The synthesis of three-dimensional (3D) polydopamine-functioned carbonyl iron powder@polypyrrole (CIP@PPy) aerogel composites for excellent microwave absorption. *Synth. Met.* **2015**, *210*, 156–164.

6. Wang, H.; Ma, N.; Yan, Z.; Deng, L.; He, J.; Hou, Y.; Jiang, Y.; Yu, G. Cobalt/polypyrrole nanocomposites with controllable electromagnetic properties. *Nanoscale* **2015**, *7*, 7189–7196.
7. Guo, J.; Song, H.; Liu, H.; Luo, C.; Ren, Y.; Ding, T.; Khan, M.A.; Young, D.P.; Liu, X.; Zhang, X.; et al. Polypyrrole-interface-functionalized nano-magnetite epoxy nanocomposites as electromagnetic wave absorbers with enhanced flame retardancy. *J. Mater. Chem. C* **2017**, *5*, 5334–5344.
8. Liu, J.; Wang, Z.; Rehman, S.; Bi, H. Uniform core-shell PPy@carbon microsphere composites with a tunable shell thickness: The synthesis and their excellent microwave absorption performances in the X-band. *RSC Adv*. **2017**, *7*, 53104–53110.
9. Shen, J.; Chen, K.; Li, L.; Wang, W.; Jin, Y. Fabrication and microwave absorbing properties of (Z-type barium ferrite/silica)@polypyrrole composites. *J. Alloy. Compd*. **2014**, *615*, 488–495.
